# Exploring Blood Cell Count-Derived Ratios as Practical Diagnostic Tools for Scabies in Vulnerable Populations

**DOI:** 10.3390/jpm14040373

**Published:** 2024-03-30

**Authors:** Hoang Thao Giang Nguyen, Ha Long Hai Le, Hoang Viet Nguyen, Huyen My Le, Huy Luong Vu, Pleiades T. Inaoka, Ota Tetsuo, Quoc Trung Ly, J. Luis Espinoza

**Affiliations:** 1 Faculty of Health Sciences, Kanazawa University, Kanazawa 920-0942, Ishikawa, Japan; nhtgiang@stu.kanazawa-u.ac.jp (H.T.G.N.); inaoka@mhs.mp.kanazawa-u.ac.jp (P.T.I.);; 2 Department of Biochemistry, Hematology and Immunology, National Hospital of Dermatology and Venereology, Hanoi 100000, Vietnam; 3 Faculty of Medical Technology, Hanoi Medical University, Hanoi 116001, Vietnam; hoangviet@hmu.edu.vn; 4 Department of Laser and Skin Care, National Hospital of Dermatology and Venereology, Hanoi 116001, Vietnam; 5 Soctrang Hospital for Women and Children, Soc Trang 950000, Vietnam; lqtrung@bvchuyenkhoasannhist.com.vn

**Keywords:** scabies, blood cell count, differential diagnosis, inflammation marker, eosinophilia

## Abstract

Scabies is a neglected tropical disease and represents a considerable global burden. Although consensus diagnostic criteria for scabies have been recently published, diagnosing scabies infestation remains challenging in clinical practice. We investigated the diagnostic utility of complete blood cell count (CBC) and CBC-derived ratios obtained at diagnosis in a set of 167 patients who are Vietnamese with confirmed scabies. These parameters were compared with those of patients with dermatophytosis (N = 800) and urticaria (N = 2023), two diseases frequent in Vietnam, which can present with similar skin manifestations to scabies and tend to pose a diagnostic challenge in vulnerable populations. Our analysis revealed that white blood cell, monocyte, and eosinophil counts were significantly higher among patients with scabies than the other two diseases. Similarly, the monocyte-to-lymphocyte ratio (MLR) and eosinophil-to-lymphocyte ratio (ELR) were significantly higher among patients with scabies. The optimal cut-off values to distinguish scabies from dermatophytosis and urticaria were 0.094 for ELR (sensitivity: 74.85%, specificity: 70.7%) and 0.295 for MLR (sensitivity: 52.69%, specificity: 73.54%). CBC, ELR, and MLR are low-cost and easily calculated parameters that may be helpful for the diagnosis of scabies.

## 1. Introduction

Scabies is a contagious skin disease caused by the mite *Sarcoptes scabiei var. hominis*, an obligate ectoparasite that infects the epidermis of the human skin. Clusters of scabies are common among vulnerable populations in overcrowded places such as prisons, refugee camps, and nursing homes. Cases of scabies can be found in every country, but the disease is particularly prevalent in many low-income tropical countries, where children and older individuals are particularly vulnerable. Similarly, communities living in developed countries are also susceptible to scabies infestations, such as in institutional outbreaks or small epidemics under war conditions or during natural disasters. Every year, more than 200 million people worldwide are affected by scabies at any time, and thus, due to its considerable global burden, scabies is included in the WHO portfolio of neglected tropical diseases [1]. In Vietnam, scabies is endemic, with a high prevalence in many settings. According to a large-scale epidemiologic investigation of skin diseases in 10 Vietnamese provinces, scabies was detected in 13% of the overall population tested and in 30% of children under 15 [2].

The symptoms of scabies typically begin 4–6 weeks after infestation and may include severe itch, often worse at night, itchy lines, and papules predominantly on the fingers, wrists, arms, legs, and belt area. Many of these symptoms may resemble those caused by other acute skin diseases, including dermatophytosis and urticaria. Importantly, due to the high humidity and defined environmental temperatures in tropical countries like Vietnam, superficial fungal infections such as dermatophytosis are common [3], and considering the high prevalence of urticaria worldwide [4], the diagnosis of scabies may be challenging, especially in vulnerable populations. According to the consensus criteria recently developed by the International Alliance for the Control of Scabies (IACS), the diagnosis of scabies includes defined levels of diagnostic certainty: “confirmed scabies”, which relies on the direct visualization of the mite or its products; “clinical scabies” (level B); and “suspected scabies” (level C), based on clinical assessment of signs and symptoms [5]. Nevertheless, in the clinical setting, misdiagnosis is not rare, which can result in delayed or inappropriate treatment, increasing the risk of outbreaks. In addition, untreated scabies is associated with skin irritation, excessive itching, scratching, and ultimately, secondary bacterial infections (most often caused by *Streptococcus pyogenes* and/or *Staphylococcus aureus*) in the skin, which, along with the impaired immunity associated with the disease, increase the risk of developing acute systemic infections [1].

Complete blood cell count (CBC) is a rapid and low-cost laboratory test broadly used in clinical practice. CBC parameters may indicate a diagnosis or point to a condition requiring more testing. In addition, some CBC-derived ratios, such as the neutrophil-to-lymphocyte ratio (NLR) and monocyte-to-lymphocyte ratio (MLR), may reflect systemic inflammatory responses with the potential to serve as disease biomarkers or tools for differential diagnosis of conditions with comparable clinical symptoms, but distinct etiologies [6].

Scabies has been reported to have a higher incidence among males and children younger than 15 [7,8,9,10]. Males often engage in more close physical contact and have a higher likelihood of participating in skin-to-skin contact, facilitating scabies mite transmission. Children’s close interaction in crowded spaces such as schools and daycare facilities also contributes to the higher prevalence of scabies in this demographic. Dermatophytosis also showed a male predominance [11] and is more commonly found in adults and immunocompromised patients [12]. Men have been reported to have a higher risk of developing dermatophytosis than women, which may be due to their frequent exposure to humid environments by sweating during physical exercises and wearing clothing with low ventilation [13]. Urticaria, however, has been reported to be more prevalent in women [14], which has been attributed to the modulation of immune function by estrogen and progesterone [15,16]. This is supported by the increased susceptibility to autoimmune and inflammatory conditions associated with sex hormone fluctuations, namely endocrinopathy, menstrual cycle, pregnancy, and menopause, which are also implicated in the severity of some diseases [17].

Using key demographic data and CBC values derived from blood samples obtained at diagnosis from patients treated at the outpatient clinic of the department of dermatology of a university hospital, this study investigated the utility of CBC values and various CBC-derived ratios as diagnostic tools to distinguish scabies from dermatophytosis and urticaria, which are two conditions frequently diagnosed in the studied population.

## 2. Materials and Methods

### 2.1. Patients and Study Design

**Figure 1:**
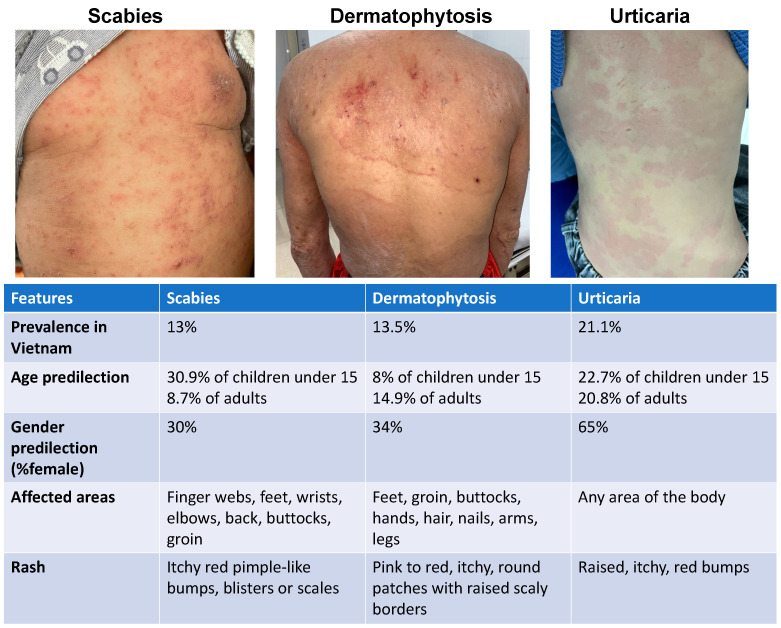
Comparison of scabies, dermatophytosis, and urticaria characteristics. The images at the top are representative skin lesions of patients who are Vietnamese with scabies, dermatophytosis, and urticaria, respectively. Key epidemiological and clinical aspects of each disease are indicated in the bottom panel.

This study retrospectively analyzed 2990 patients at the Department of Dermatology at Hanoi Medical University Hospital in 2022, including 167 patients with scabies, 800 with dermatophytosis, and 2023 with urticaria. As a control group, the study also included a total of 1943 healthy individuals whose physical examination and blood tests, including CBC, were obtained as part of a regular annual health checkup and served to determine the normal CBC values and the CBC-derived ratios in the population who are Vietnamese. We focused on these three skin diseases (scabies, dermatophytosis, and urticaria), given the presence of multiple similarities of these conditions in terms of clinical characteristics, age, and distribution in the studied population (Figure 1), as well as for the reported alterations in CBC parameters, including eosinophilia and neutrophilia, associated with these conditions [2,8,18,19].

The diagnosis of scabies was determined by wet mount and dermoscopy. Dermatophytosis was confirmed by wet mount and culture with morphology identification. The study was conducted in accordance with the Declaration of Helsinki, and informed consent to participate in the study was obtained from participants. Ethical approval for this study was waived by the Ethics Committee of Hanoi Medical University as it utilized an anonymized dataset. Key demographic and laboratory data, including patient age, gender, and diagnosis, as well as the values of the CBC of patients included in the study, were retrieved from electronic medical records available in our institution.

### 2.2. Blood Analysis and Calculations

Blood samples of all individuals were collected at diagnosis and analyzed in a DxH 600 Hematology Analyzer (Beckman Coulter Inc., Brea, CA, USA) to determine the blood cell count parameters. The neutrophil-to-lymphocyte ratio (NLR), monocyte-to-lymphocyte ratio (MLR), eosinophil-to-lymphocyte ratio (ELR), basophil-to-lymphocyte ratio (BLR), and platelet-to-lymphocyte ratio (PLR) were calculated by dividing the neutrophil, monocyte, eosinophil, basophil, and platelet count by the lymphocyte count, respectively.

The normal reference values of the distinct cellular components of the CBC in the studied population are shown in Supplementary Materials. As a result, leukocytosis was defined as a white blood cell count > 11 G/L, neutrophilia with a neutrophil count > 7.7 G/L, lymphocytosis with a lymphocyte count > 4 G/L, monocytosis with a monocyte count > 0.95 G/L, eosinophilia with an eosinophil count > 0.5 G/L, and basophilia with a basophil count > 0.15 G/L.

### 2.3. Statistical Analysis

Statistical analysis was performed using *SPSS* version 25.0 (IBM Corp., Armonk, NY, USA) and *MedCalc* version 20.026 (MedCalc Software Ltd., Ostend, Belgium). The normality of values was assessed using the Kolmogorov–Smirnov test. As most data were not normally distributed, continuous variables are reported as a median with the 25–75% interquartile range (IQR). The parameters of the three diseases were compared using the Kruskal–Wallis test, incorporating the Dunn–Bonferroni post hoc test for pairwise comparison. The Mann–Whitney U test was used to compare the variables between each disease and the healthy control. Categorical variables are expressed as numbers (n) and percentages (%) and compared using the chi-squared or Fisher’s exact test. The analyses of the receiver operating characteristic (ROC) curves were conducted on complete-blood-count (CBC)-derived ratios, white blood cell counts, and white blood cell percentages. An area under the curve (AUC) greater than 0.6 was considered acceptable for diagnostic purposes [20]. The AUCs constructed by different parameters were compared using Delong’s method. The cutoff values were determined using the maximum Youden index. A *p*-value less than 0.05 was considered statistically significant.

## 3. Results

Our analysis included the CBC values and CBC-derived ratios from 1943 healthy controls and a total of 2990 patients who are Vietnamese with defined dermatological diseases (167 with scabies, 800 with dermatophytosis, and 2023 with urticaria). Figure 1 (upper panel) shows representative skin lesions observed in each of these diseases. The median age of the patients with scabies was 20 years (IQR: 14–33.75), which was significantly lower than that of the group with dermatophytosis (36 years, IQR: 21–50) and the group with urticaria (26 years, IQR: 13–40), with *p* < 0.001. Patients with scabies were predominantly male (77.2%), as was the case for those with dermatophytosis (61.3%), but not those with urticaria, where 59.7% of them were female (*p* < 0.001) (Table 1).

Compared to the dermatophytosis and urticaria groups, patients with scabies exhibited a significantly higher median white blood cell count (*p* < 0.001), monocyte count (*p* < 0.001), and eosinophil count (*p* < 0.001). Patients with scabies also showed higher neutrophil counts than patients with dermatophytosis (pairwise *p* = 0.015) and higher basophil counts than patients with urticaria (pairwise *p* = 0.001), but there was no significant difference in lymphocyte and platelet counts compared to the other two groups (*p* > 0.05 in pairwise comparisons). Consequently, the patients with scabies showed elevated medians of MLR (*p* < 0.001) and ELR (*p* < 0.001) in comparison with patients with dermatophytosis and urticaria. They also had a higher NLR (pairwise *p* = 0.016) and BLR (pairwise *p* < 0.001) than patients with urticaria, but there was no significant difference in the PLR compared to the other two groups (*p* = 0.269). Regarding the percentage of different white blood cell types, the neutrophil percentage remained the same between the three patient groups (*p* = 0.879), but the monocyte and eosinophil percentages were both elevated in patients with scabies compared to the patients with dermatophytosis and urticaria. Patients with scabies also showed a decrease in the percentage of lymphocytes in comparison with the group with dermatophytosis (pairwise *p* = 0.053) and the group with urticaria (significantly with pairwise *p* < 0.001). Patients with urticaria had significant decreases in eosinophil-related parameters (eosinophil count and percentage, ELR, and percentage of eosinophilia), as well as basophil-related parameters (basophil count and percentage, BLR) in comparison with patients with scabies and dermatophytosis. The percentage of patients with leukocytosis, neutrophilia, monocytosis, and eosinophilia was also significantly higher in the group with scabies (Table 2).

In comparison with the healthy control group, all three diseases had significantly higher white blood cell, neutrophil, and monocyte counts. The three diseases also showed elevated NLR, PLR, and MLR compared to the healthy group. It is worth noting that the lymphocyte count of the patients with scabies was not significantly different from the healthy group (*p* = 0.25). All of the eosinophil-related parameters, namely the eosinophil count and percentage, ELR, and eosinophilia percentage, of the group with urticaria were significantly decreased in comparison with the healthy control. These patients also showed a significantly lower basophil count.

**Figure 2:**
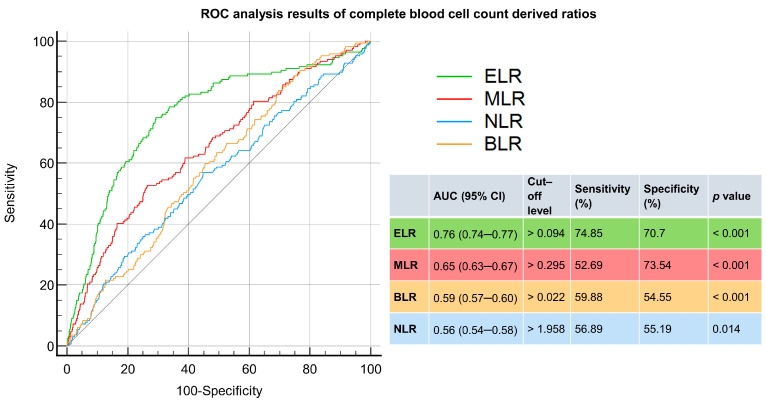
ROC analysis of complete-blood-cell-count-derived ratios to predict scabies. NLR: neutrophil-to-lymphocyte ratio; PLR: platelet-to-lymphocyte ratio; MLR: monocyte-to-lymphocyte ratio; ELR: eosinophil-to-lymphocyte ratio; BLR: basophil-to-lymphocyte ratio.

ROC analyses were conducted to identify diagnostic markers with a significant value in differentiating scabies from dermatophytosis and urticaria. The ELR had the greatest AUC value (0.76), followed by the MLR (0.65). Both the BLR and NLR had AUC values less than 0.6. The cut-off level for the ELR was 0.094, with a sensitivity and specificity of 74.85% and 70.7%, respectively. With a cut-off value of 0.295, the sensitivity and specificity of the MLR were 52.69% and 73.54%, respectively (Figure 2).

**Figure 3:**
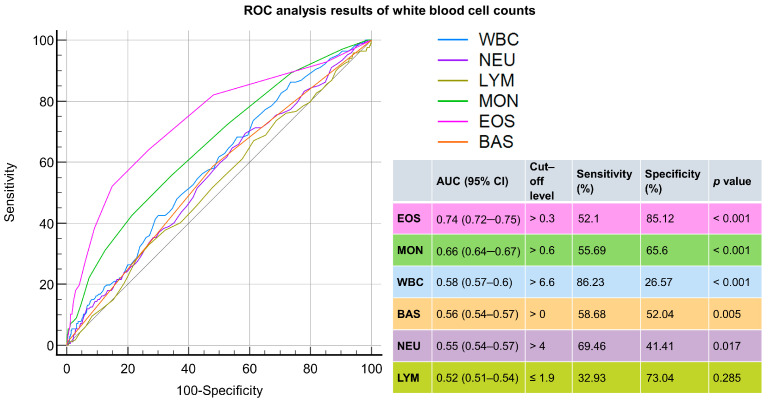
ROC analysis of white blood cell counts for distinguishing scabies from dermatophytosis and urticaria. The parameters include white blood cells (WBCs); neutrophils (NEUs); lymphocytes (LYMs); monocytes (MONs); eosinophils (EOSs); and basophils (BASs).

On the other hand, we observed that the eosinophil count and monocyte count each had an AUC of more than 0.6 in differentiating scabies from dermatophytosis and urticaria, with values at 0.74 and 0.66, respectively (Figure 3).

**Figure 4:**
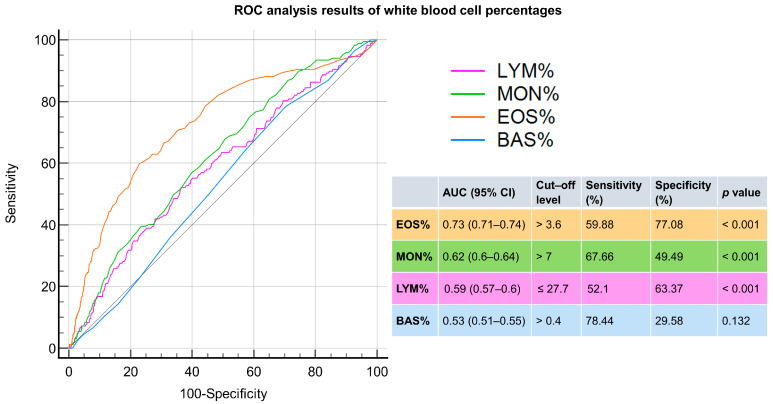
ROC analysis of white blood cell percentages for distinguishing scabies from dermatophytosis and urticaria. The parameters include the percentage of lymphocytes (LYM%), percentage of monocytes (MOM%), percentage of eosinophils (EOS%), and percentage of basophils (BAS%).

Similarly, the percentage of eosinophils and the percentage of monocytes also exhibited AUCs above 0.6 in differentiating scabies from dermatophytosis and urticaria, with values of 0.73 and 0.62, respectively (Figure 4).

**Figure 5:**
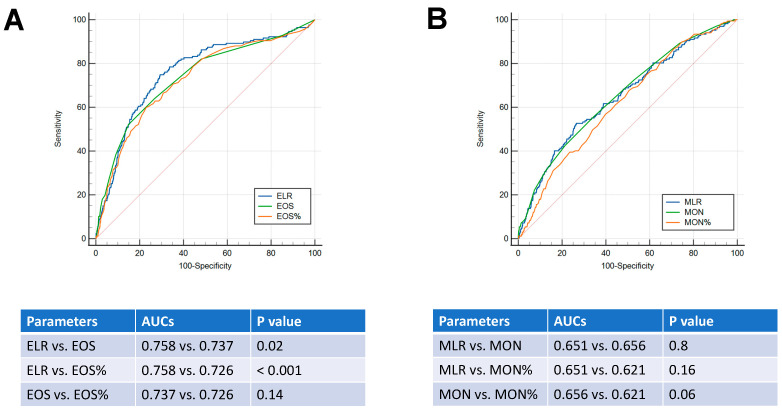
Comparison of the ROC curves for distinguishing scabies from dermatophytosis and urticaria. (**A**) Eosinophil parameters included the eosinophils to lymphocyte rate (ELR), eosinophil count (EOS), and percentage of eosinophils (EOS%). (**B**) Monocyte parameters included monocyte-related parameters, including the MLR (monocyte to lymphocyte rate), monocyte count (MON), and percentage of monocytes (MON%).

Finally, a comparison between the area under the ROC curves revealed that the ELR exhibited a significantly higher AUC than both absolute eosinophil count (*p* = 0.02) and eosinophil percentage, in differentiating scabies from dermatophytosis and urticaria (*p* < 0.001) (Figure 5A). However, all three monocyte-related parameters (MLR, monocyte count, and percentage of monocytes) did not demonstrate statistically significant AUCs when compared to each other (Figure 5B), thus substantiating the relevance of the ELR as a tool that could be helpful for the diagnosis of scabies.

## 4. Discussion

This study is the first to exhaustively examine the diagnostic utility of CBC and CBC-derived ratios in scabies. We found that white blood cell, monocyte, and eosinophil counts, as well as MLR and ELR levels were significantly higher among patients with scabies than in those with dermatophytosis or urticaria, which may reflect a more intense systemic inflammatory response associated with scabies than these diseases.

Scabies mites burrow into the epidermis and produce soluble antigens via saliva, eggs, feces, and other secretions, which diffuse into the dermis and stimulate the immune system of the host, which promotes a strong immune response characterized in the first stages of the infestation as a type I (immediate) hypersensitivity reaction against the mite and its products. Type I hypersensitivity reactions primarily involve immunoglobulin E (IgE), mast cells, and eosinophils. In the initial phase, IgE antibodies are produced in response to mite antigens and bind to the Fc receptors of mast cells and basophils. In the skin lesions of patients with scabies, increases in mast cell and basophil infiltration have been observed [21,22]. Subsequently, crosslinking of the antigens to these cells results in the degranulation of the cells and the release of histamine, prostaglandin, leukotrienes, and tryptase, which are implicated in the itching mechanism of the disease through the activation of the histaminergic receptors H1 and H4 [23,24]. Cytokines released from mast cells also recruit eosinophils and neutrophils to the site of reaction over several hours; this late-phase reaction is responsible for the tissue injury [25]. In addition to the pivotal role of the type I hypersensitivity reaction in the pathogenesis of scabies, accumulating evidence indicates that a type IV (delayed) hypersensitivity reaction is also implicated in the pathogenesis of scabies [26]. This type IV hypersensitivity, which occurs up to four weeks after the initial infestation, can manifest as eczematous or erythematous papules and vesicles [24,27]. The molecular mechanisms underlying this reaction involve the stimulation of T cells by antigen-presenting cells, which promotes cytokine secretion and the additional recruitment of other immune cells and manifests clinically as pruritus and skin lesions [28]. Histological analysis has documented the infiltration of CD4+ T cells and CD8+ T cells in the skin of patients with scabies, thus supporting the implication of the cell-mediated immune response to mites in the pathogenesis of the disease [29]. Regarding the specific lymphocyte populations involved in this process, some studies have reported that the number of T and B lymphocytes and T cell subsets in the blood of patients with scabies was within normal ranges [26,30]; however, the predominant type of T lymphocytes in the skin lesion was different depending on the severity of the disease, suggesting a selective infiltration. CD4^+^ T cells were demonstrated to be prevalent in the skin of ordinary patients with scabies, while crusted scabies lesions had an increased number of CD8^+^ T cells compared to minimal CD4^+^ cell count [30,31].

Eosinophil infiltration with or without eosinophilia is a frequent finding in a large number of skin diseases, and since eosinophils contribute to host defense against invading pathogens, participate in the regulation of other immune cells, and directly or indirectly cause pruritus, tissue damage, and tissue remodeling, they are implicated in the pathogenesis of various skin diseases. Histological analysis of skin infected by the scabies mite resembles a chronic allergic reaction and is infiltrated by mainly eosinophils, along with Langerhans cells, T cells, monocytes, macrophages, and mast cells [32,33]. Scabies, like other parasitic infections, can cause eosinophilia, and it is plausible that the degree of eosinophilia could be associated with the severity of the scabies infestation. For example, in crusted scabies, also known as Norwegian scabies, which is a severe form of the disease with extensive hyperkeratotic skin lesions, crusting, and scaling, eosinophilia has been reported in more than 50% of patients [34], and eosinophilia has been frequently found in patients with scabies co-existing with other dermatoses like psoriasis [35,36]. Importantly, in our search of the literature, we did not find studies investigating the relevance of CBC and CBC-derived ratios in patients with scabies. In our study, the analysis of ROC curves revealed that the ELR was a more reliable marker than the absolute eosinophil count or the percentage of eosinophils to distinguish scabies from urticaria and dermatophytosis. Since the ELR reflects the abundance of eosinophils relative to lymphocytes, it may provide more nuanced information because, in patients with scabies, the numbers of circulating lymphocytes were similar to those in healthy individuals and in those with dermatophytosis and urticaria.

In this study, we observed that patients with urticaria had lower eosinophil and basophil counts than those with scabies or dermatophytosis. This finding is important since urticaria is a well-known cause of eosinophilia and basophilia; however, previous studies have also shown that, in patients with chronic spontaneous urticaria, a decrease of eosinophils and basophils in the peripheral blood is found due to the infiltration of these cells into the skin lesions [37,38]. Another mechanism in these patients involves the autoimmune destruction of eosinophils and basophils in the blood, resulting in a lack of these cells in the peripheral blood, bone marrow, and skin [39].

The immunopathogenesis of dermatophytosis involves the interplay of both the innate and acquired (humoral- and cell-mediated) immune systems, which not only recognize and eradicate the intruding fungi, but also contribute to the local symptoms associated with this disease [3]. Neutrophils, dendritic cells, keratinocytes, macrophages, and various pro-inflammatory cytokines such as IL-6, IL-17, and TNF are key components of the innate immune response against the fungal infection, while cell-mediated immunity increases epidermal proliferation, promotes cell turnover, and ultimately, facilitates the elimination of fungi [40]. Interestingly, a predominant Th1-type immune response facilitates the elimination of fungi, while a Th2 immune response predisposes to infection or results in an allergic response [3]. On the other hand, the relevance of circulating leukocytes, including eosinophilia, in patients with dermatophytosis has not been extensively studied, as most research has been focused on the local immune response to the disease and the laboratory methods to confirm the presence of the infective agent [41]. We found only one study that comprehensively investigated the presence of eosinophilia in the peripheral blood of patients with this condition. In the study, which was conducted in India and included a total of 282 cases, eosinophilia was documented in 27% of cases. Interestingly, no peripheral eosinophilia was observed in the remaining 23 (8%) cases of other cutaneous fungal infections [42]. In our study, eosinophilia was less frequent, being found in 87 (11%) of the 800 patients with dermatophytosis. The reason for this apparent discrepancy is currently unclear, but it could be associated with geographical or genetic differences between the studied populations, the fungal strains involved, or differences in the severity of the cases included in both studies.

The ratios between myeloid cells and lymphocytes (NLR, MLR, ELR, BLR) mirror the balance between the innate and adaptive aspects of the immune system. These elevated ratios in patients with scabies were resulted from higher counts of neutrophils, monocytes, eosinophils, and basophils, while the lymphocyte count was similar between scabies and the other two diseases. This demonstrated a strong innate response to the scabies mites with the recruitment of granulocytes and monocytes. In addition, the lymphocyte count being not considerably higher may reflect a temporary delay in the activation of adaptive immune response, allowing the mite to survive longer in the host [43]. This postponement could stem from *S. scabiei*’s ability to evade the complement cascade through the secretion of scabies mite serine protease inhibitors SMSB3 and SMSB4 [44].

Incorporating CBC-derived ratios into the diagnostic process has been shown to not only enhance the accuracy of distinguishing skin disorders with different etiologies, but also contribute to personalized medicine approaches. By monitoring the inflammatory markers, health providers may have the ability to tailor treatment strategies based on the immune profile of an individual. In clinical practice, this could help clinicians optimize therapeutic outcomes and improve patient care in a more precise and effective manner. For example, the NLR, PLR, neutrophil-to-monocyte ratio, and systemic immune-inflammation index were found to be useful biomarkers in tracking the response to treatment with TNF-α inhibitors in patients with psoriasis [45]. As for scabies, TLR8, IL-17, and IL-23 have been shown to be promising targets for immunotherapy to treat crusted scabies, alongside antiparasitic drugs [30,46]. CBC-derived ratios and other systematic inflammatory indices could be potential markers for diagnosing the severity and monitoring the treatment of this debilitating form of scabies.

There are some limitations associated with this study, including the lack of detailed information regarding the severity of scabies and other skin conditions in the patients included in the study, which restricts our ability of draw precise conclusions about the variability of the immune response in different stages or the intensity of the condition. Furthermore, the unavailability of other demographic variables and baseline data impeded us from performing additional studies such as multivariate analyses, which would have been instrumental in investigating the diagnostic markers in diverse clinical scenarios and subgroups. However, our study also has several strengths that support its clinical relevance. This is the first study to comprehensively analyze the role of the CBC and CBC-derived ratios as a potential diagnostic tool for scabies, and to the best of our knowledge, our study includes the largest dataset analyzed so far of three dermatological diseases (167 with scabies, 800 with dermatophytosis, and 2023 with urticaria) that share various clinical manifestations, and we have identified specific findings associated with scabies. In addition, our data were validated by the inclusion of 1943 healthy controls derived from a very homogeneous population, which allowed us to determine, with a reasonably high level of accuracy, the normal reference values of the CBC and CBC-derived ratios in the healthy population and, ultimately, facilitate the interpretation of any variations of these values in the three diseases included in the study. Further studies could benefit from incorporating the severity of scabies as a key variable and explore the correlation between the intensity with subsets of T lymphocytes. This could provide deeper insights into the immunopathology of scabies and inform more targeted therapeutic strategies. In conclusion, after analyzing a large dataset of patients who are Vietnamese (n = 2990) and 1943 healthy controls, we found that white blood cell, monocyte, and eosinophil counts, as well as the MLR and ELR levels are significantly higher among patients with scabies than in those with dermatophytosis or urticaria. These findings in the blood may reflect a more intense systemic inflammatory response associated with scabies than these diseases. Therefore, patients with suspected scabies could benefit from the CBC and CBC-derived ratios as inexpensive and simple parameters that could aid clinicians in the diagnosis of scabies.

## Figures and Tables

**Table 1:** Demographic characteristics of the populations with a disease.

Variable	Scabies	Dermatophytosis	Urticaria	*p*-Value
	(N = 167)	(N = 800)	(N = 2023)	
Age, years; median (IQR)	20 (14–33.75)	36 (21–50)	26 (13–40)	<0.001
Gender				<0.001
Male; n (%)	129 (77.2%)	490 (61.3%)	815 (40.3%)	
Female; n (%)	38 (22.8%)	310 (38.8%)	1208 (59.7%)	

**Table 2:** Complete blood cell counts parameters and ratios in the studied populations.

Variable	Scabies	Dermatophytosis	Urticaria	Healthy Control	*p*-Value
	(N = 167)	(N = 800)	(N = 2023)	(N = 1943)	
CBC parameters, G/L; median (IQR)					
White blood cell count	8.8 (7.3–10.6)	7.75 (6.5–9.4) ^a,b^	8.2 (6.6–10.2) ^a^	6.88 (5.9–8)	<0.001 *
Neutrophil count	4.9 (3.7–6.5)	4.4 (3.5–5.7) ^a^	4.5 (3.3–6.3)	3.64 (2.97–4.5)	0.015 *
Lymphocyte count	2.3 (1.8–2.9) ^c^	2.3 (1.8–2.8) ^b^	2.4 (1.9–3.2)	2.42 (2–2.85)	<0.001 *
Monocyte count	0.7 (0.5–0.9)	0.6 (0.5–0.7) ^a^	0.6 (0.4–0.7) ^a^	0.49 (0.4–0.59)	<0.001 *
Eosinophil count	0.4 (0.2–0.6)	0.2 (0.1–0.3) ^a,b^	0.1 (0.1–0.2) ^a^	0.17 (0.1–0.27)	<0.001 *
Basophil count	0.1 (0–0.1)	0.1 (0–0.1) ^b^	0 (0–0.1) ^a^	0.03 (0.02–0.05)	<0.001 *
Platelet count	264 (226.5–326.75)	258 (221–303) ^b,c^	274 (232.25–323)	255 (224–290)	<0.001 *
White blood cell percentage, %; median (IQR)					
Neutrophil %	57.7 (50.6–67.3)	57.3 (50.9–64.1)	57.8 (49.4–66.6)	53.6 (48.4–59.4)	0.879
Lymphocyte %	27.5 (20.2–35.5)	30.4 (24.4–36.4) ^b^	31.7 (24.2–39.3) ^a^	35.4 (30.3–40.6)	<0.001 *
Monocyte %	7.8 (6.6–9.5)	7.4 (6.2–8.7) ^a,b^	6.9 (5.7–8.3) ^a^	7 (6.1–8.2)	<0.001 *
Eosinophil %	4.3 (2.3–6.8)	2.6 (1.5–4.4) ^a,b^	1.6 (0.8–3) ^a^	2.4 (1.5–3.9)	<0.001 *
Basophil %	0.6 (0.5–0.8)	0.7 (0.5–0.9) ^b^	0.6 (0.4–0.8) ^a^	0.5 (0.3–0.7)	<0.001 *
CBC-derived ratios; median (IQR)					
NLR	2.09 (1.45–3.24)	1.88 (1.41–2.62)	1.83 (1.27–2.73) ^a^	1.51 (1.2–1.95)	0.007 *
PLR	114.1 (90.6–152.2)	111.1 (88.3–141.8)	110 (85.9–141)	105.6 (86.8–131)	0.269 *
MLR	0.299 (0.21–0.408)	0.249 (0.191–0.329) ^a,b^	0.22 (0.17–0.292) ^a^	0.201 (0.165–0.249)	<0.001 *
ELR	0.151 (0.091–0.21)	0.088 (0.048–0.151) ^a,b^	0.051 (0.027–0.088) ^a^	0.069 (0.043–0.113)	<0.001 *
BLR	0.023 (0.017–0.031)	0.023 (0.017–0.032) ^b^	0.019 (0.013–0.027) ^a^	0.014 (0.01–0.019)	<0.001 *
Blood cytoses; n (%)					
Leukocytosis	35 (21%)	97 (12.1%)	355 (17.5%)	63 (3.2%)	<0.001 **
Neutrophilia	27 (16.2%)	66 (8.3%)	274 (13.5%)	36 (1.9%)	<0.001 **
Lymphocytosis	13 (7.8%)	28 (3.5%)	225 (11.1%)	47 (2.4%)	<0.001 **
Monocytosis	37 (22.2%)	54 (6.8%)	153 (7.6%)	26 (1.3%)	<0.001 **
Eosinophilia	45 (27%)	87 (11%)	80 (4%)	113 (5.8%)	<0.001 **
Basophilia	3 (1.8%)	7 (0.9%)	17 (0.8%)	0 (0%)	<0.001 **

Abbreviations: NLR: neutrophil-to-lymphocyte ratio; PLR: platelet-to-lymphocyte ratio; MLR: monocyte-to-lymphocyte ratio; ELR: eosinophil-to-lymphocyte ratio; BLR: basophil-to-lymphocyte ratio. * indicates the *p* value of the Kruskal–Wallis test to compare the three patient groups. ** indicates the *p*-value of the chi-squared or Fisher’s exact test to compare the four groups. ^a^ denotes a *p* < 0.05 in the pairwise comparison with the group with scabies. ^b^ denotes a *p* < 0.05 in the pairwise comparison between the groups with dermatophytosis and urticaria. ^c^ denotes a *p* > 0.05 in the pairwise comparison with the healthy control group.

## Data Availability

The data are available up reasonable request from the corresponding author.

## Supplementary Materials

The following supporting information can be downloaded at: https://www.mdpi.com/article/10.3390/jpm14040373/s1.
