# Supplementary material for: Exploring Blood Cell Count-Derived Ratios as Practical Diagnostic Tools for Scabies in Vulnerable Populations
Source: J Pers Med. 2024 Mar 30;14(4):373. doi: 10.3390/jpm14040373 (PMC11050978; doi:10.3390/jpm14040373)
Supplement: Supplementary file 1 [file jpm-14-00373-s001.zip › jpm-2894561-supplementary.pdf]

Hematology Reference Ranges

Test	Method	Sex	Criteria	Reference Range	Units
**WBC**	DXH600		**0-1 Day**	9.0 - 30.0	$x10^3$ / uL
			**1-4 Days**	9.4 - 34.0	
			**4 Days - 1 Month**	5.0 - 20.0	
			**1 Month - 6 Months**	4.0 - 19.5	
			**6 Months-2 Years**	6.0 - 17.0	
			**2-6 Years**	5.0 - 15.5	
			**6-18 Years**	4.5 - 13.5	
		Male	**> or = 18 Years**	4.5 - 11.0	
		Female	**> or = 18 Years**	4.5 - 11.0	

Test	Method	Sex	Criteria	Reference Range	Units
**RBC**	DXH600		**0-1 Day**	3.90 - 5.90	$x10^6$ / uL
			**1-3 Days**	3.90 - 5.90	
			**4 Days - 1 Month**	3.90 - 5.90	
			**1 Month - 6 Months**	3.30 - 5.30	
			**6 Months-2 Years**	3.90 - 4.80	
			**2-6 Years**	3.70 - 4.90	
			**6-18 Years**	3.80 - 5.60	
		Male	**> or = 18 Years**	4.30 - 5.80	
		Female	**> or = 18 Years**	3.80 - 5.20	

Test	Method	Sex	Criteria	Reference Range	Units
**Hemoglobin (Hgb)**	DXH600		**0-1 Day**	19.0 - 30.0	g/dL
			**1-3 Days**	14.5 - 22.5	
			**4 Days - 1 Month**	13.4 - 19.8	
			**1-2 Months**	10.7 - 17.1	g/dL
			**2-6 Months**	9.4 - 13.0	
			**6 Months-2 Years**	10.5 - 13.5	
			**2-6 Years**	11.5 - 13.5	
			**6-12 Years**	11.5 - 15.5	
		Male	**12-18 Years**	13.0 -16.0	g/dL
		Female	**12-18 Years**	12.0 - 16.0	
		Male	**18 Years and Older**	13.5 - 17.5	
		Female	**18 Years and Older**	12.0 - 16.0	

Test	Method	Sex	Criteria	Reference Range	Units
**Hematocrit (Hct)**	DXH600		**0-4 Days**	45 - 67	%
			**4 Days - 1 Month**	41 - 65	
			**1-2 Months**	33 - 55	
			**2-6 Months**	28 - 42	
			**6 Months-2 Years**	33 - 39	
			**2-6 Years**	34 - 40	
			**6-12 Years**	35 - 45	
		Male	**12-18 Years**	36 - 50	
		Female	**12-18 Years**	37 - 45	
		Male	**18 Years and Older**	41 - 53	
		Female	**18 Years and Older**	36 - 46	

Test	Method	Sex	Criteria	Reference Range	Units
**MCH**	DXH600		**0-1 Day**	30 - 37	pg
			**1-3 Days**	30 - 37	
			**4 Days - 1 Month**	30 - 37	
			**1-6 Months**	29 - 36	
			**6 Months-2 Years**	24 - 30	
			**2-6 Years**	25 - 31	
			**6-18 Years**	25 - 34	
		Male	**> or = 18 Years**	27 - 35	
		Female	**> or = 18 Years**	27 - 35	

Test	Method	Sex	Criteria	Reference Range	Units
**MCHC**	DXH600		**0-4 Days**	29.0 - 37.0	g/dL
			**4 Days - 1 Month**	28.1 - 34.7	
			**1-2 Months**	28.1 - 35.5	
			**2-6 Months**	28.3 - 35.3	
			**6 Months-2 Years**	30.0 - 36.0	
			**2-6 Years**	31.0 - 37.0	
			**6-12 Years**	31.0 - 37.0	
		Male	**12-18 Years**	31.0 - 37.0	
		Female	**12-18 Years**	31.0 - 37.0	
		Male	**18 Years and Older**	31.0 - 37.0	
		Female	**18 Years and Older**	31.0 - 37.0	

Test	Method	Sex	Criteria	Reference Range	Units
**MCV**	DXH600		**0-4 Days**	95 - 121	fL
			**4 Days - 1 Month**	88 - 122	
			**1-2 Months**	91 - 111	
			**2-6 Month**	84 - 106	
			**6 Months-2 Years**	70 - 86	
			**2-6 Years**	75 - 87	
			**6-12 Years**	77 - 95	
		Male	**12-18 Years**	78 - 98	
		Female	**12-18 Years**	78 - 98	
		Male	**18 Years and Older**	80 - 100	
		Female	**18 Years and Older**	80 - 100	

Test	Method	Sex	Criteria	Reference Range	Units
**Platelet Count**	DXH600		**0-3 Days**	150 - 350	x10^3 / uL
			**4 Days- 1 Month**	150 - 350	
			**1-2 Months**	150 - 350	
			**2-6 Months**	150 - 350	
			**6 Months-2 Years**	150 - 350	
			**2-6 Years**	150 - 350	
			**6-12 Years**	150 - 350	
		Male	**12-18 Years**	150 - 350	
		Female	**12-18 Years**	150 - 350	
		Male	**> or = 18 Years**	150 - 350	

		Female	**> or = 18 Years**	150 - 350	

Test	Method	Sex	Criteria	Reference Range	Units
**RDW-CV**	DXH600		**0-7 months**	14.9 - 18.7	
			**> 7 months**	11.6 - 14.8	

Test	Method	Sex	Criteria	Reference Range	Units %
**Automated Differential**	DXH600	Male	**NEUT%**	see below	%
			**LYMPH%**	see below	
			**MONO%**	4.7 - 13.9	
			**EOS%**	0.0 - 6.1	
			**BASO%**	0.0 - 1.2	
		Female	**NEUT%**	see below	
			**LYMPH%**	see below	
			**MONO%**	3.7 - 11.9	
			**EOS%**	0.0 - 5.4	
			**BASO%**	0.0 - 1.5	
**Neutrophil**			**Criteria**	**x 10^3/uL**	**%**
			0 Day	6.0 - 28.0	68 (54.4 - 81.6)
			24 Hour	5.0 - 21.0	61 (48.8 - 73.2)
			1 Week	1.5 - 10	45 (36 - 54)
			2 Week	1 - 9.5	40 (32 - 48)
			1 Month	1 - 8.5	35 (28 - 42)
			6 Month	1 - 8.5	32 (25.6 - 38.4)
			1 Year	1.5 - 8.5	31 (24.8 - 37.2)
			2 Year	1.5 - 8.5	33 (26.4 - 39.6)
			4 Year	1.5 - 8.5	42 (33.6 - 50.4)
			6 Year	1.5 - 8	51 (40.8 - 61.2)
			8 Year	1.5 - 8	53 (42.4 - 63.6)
			10 Year	1.5 - 8.5	54 (43.2 - 64.8)
			16 Year	1.8 - 8	57 (45.6 - 68.4)
			21-older	1.8 - 7.7	59 (47.2 - 70.8)

			Criteria	x 10^3/uL	%
**Lymphocyte**			0 Day	2 - 11	24 (19.2 - 28.8)
			24 Hour	2 - 11.5	31 (24.8 - 37.2)
			1 Week	2 - 17	41 (32.8 - 49.2)
			2 Week	2 - 17	48 (38.4 - 57.6)
			1 Month	2.5 - 16.5	56 (44.8 - 67.2)
			6 Month	4 - 13.5	61 (48.8 - 73.2)
			2 Year	3 - 9.5	59 (47.2 - 70.8)
			4 Year	2 - 8	50 (40 - 60)
			6 Year	1.5 - 7	42 (33.6 - 50.4)
			8 Year	1.5 - 6.8	39 (31.2 - 46.8)
			10 Year	1.5 - 6.5	38 (30.4 - 45.6)
			16 Year	1.2 - 5.2	35 (28 - 42)
			21-older	1 - 4.8	34 (27.2 - 40.8)
